# Leave events among Aboriginal and Torres Strait Islander people: a systematic review

**DOI:** 10.1186/s12889-022-13896-1

**Published:** 2022-08-05

**Authors:** J Coombes, K Hunter, K Bennett-Brook, B Porykali, C Ryder, M Banks, N Egana, T Mackean, S Sazali, E Bourke, C Kairuz

**Affiliations:** 1 The George Institute for Global Health, Newtown, Australia; 2 The University of New South Wales, Sydney, Australia; 3 College of Medicine and Public Health, Flinders University, Adelaide, Australia; 4 Australian Commission On Safety and Quality in Health Care, Sydney, Australia

**Keywords:** Aboriginal, Leave events, Quality of care, Torres Strait Islander

## Abstract

**Background:**

Leave events are a public health concern resulting in poorer health outcomes. In Australia, leave events disproportionally impact Aboriginal and Torres Strait Islander people. A systematic review was conducted to explore the causes of leave events among Aboriginal and Torres Strait Islander people and strategies to reduce them.

**Methods:**

A systematic review was conducted using Medline, Web of Science, Embase and Informit, a database with a strong focus on relevant Australian content. Additionally, we examined the references of the records included, and performed a manual search using Google, Google scholar and the Australia’s National Institute for Aboriginal and Torres Strait Islander Health Research. Two independent reviewers screened the records. One author extracted the data and a second author reviewed it. To appraise the quality of the studies the Mixed Methods Appraisal Tool was used as well as the Aboriginal and Torres Strait Islander Quality Appraisal Tool. A narrative synthesis was used to report quantitative findings and an inductive thematic analysis for qualitative studies and reports.

**Results:**

We located 421 records. Ten records met eligibility criteria and were included in the systematic review. From those, four were quantitative studies, three were qualitative studies and three reports. Five records studied data from the Northern Territory, two from Western Australia, two from New South Whales and one from Queensland. The quantitative studies focused on the characteristics of the patients and found associations between leave events and male gender, age younger than 45 years and town camp residency. Qualitative findings yielded more in depth causes of leave events evidencing that they are associated with health care quality gaps. There were multiple strategies suggested to reduce leave events through adapting health care service delivery. Aboriginal and Torres Strait Islander representation is needed in a variety of roles within health care provision and during decision-making.

**Conclusion:**

This systematic review found that multiple gaps within Australian health care delivery are associated with leave events among Aboriginal and Torres Strait Islander people. The findings suggest that reducing leave events requires better representation of Aboriginal and Torres Strait Islander people within the health workforce. In addition, partnership with Aboriginal and Torres Strait Islander people is needed during the decision-making process in providing health services that meet Aboriginal and Torres Strait Islander cultural needs.

**Supplementary Information:**

The online version contains supplementary material available at 10.1186/s12889-022-13896-1.

## Background

Leave events, Discharge Against Medical Advice (DAMA) or self-discharge, describe events where a patient leaves a health service before being seen by a health professional or before discharge by their clinician [1]. These are a public health concern [2, 3] given the increased risk of unplanned hospital readmissions and mortality [1, 4, 5]. There are differences between the terminology used by States and Territories for leave events. Supplementary file 1.

The Australian Institute of Health and Welfare reported age-standardised leave event rates of 16 per 1,000 in 2016–2017 and between July 2015 and June 2017, 19,900 Aboriginal and Torres Strait Islander hospital patients took their own leave from hospital nationally [6]. In addition, more recent unpublished data provided by the Australian Commission on Safety and Quality in Health Care evidenced that for the year 2018/19, 1% of all hospitalisations for non-Indigenous Australians were DAMA, whilst for Aboriginal and Torres Strait Islander people DAMA accounted for 4.19% of all hospitalisations. Of all leave events for hospital admitted patients, 23.2% are patients who identified as Aboriginal or Torres Strait Islander people. Discharge from hospital against medical advice occurs at a rate four times greater for Aboriginal and Torres Strait Islander patients as compared to non-Indigenous Australians.

Leave events are associated with patient dissatisfaction and studies have shown that negative hospital experiences can result in patients deciding to leave hospital against medical advice [7]. Thus, leave events can be interpreted as an indirect measure of patient dissatisfaction [8]. In an Australian context, this reflects the extent to which health services are responsive to Aboriginal and Torres Strait Islander people’s needs [9]. The ongoing health gap in multiple health indicators between Aboriginal and Torres Strait Islander people and other Australians reflects the continuous failure of Australian health services to meet Aboriginal and Torres Strait Islander health needs [10].

Understanding the causes of leave events among Aboriginal and Torres Strait Islander people is important to develop and implement culturally safe mechanisms for health services to better meet Aboriginal and Torres Strait Islander peoples’ health needs. Given the higher burden of leave events among Aboriginal and Torres Strait Islander people, the Australian Commission on Safety and Quality in Health Care appointed The George Institute to conduct a systematic review analysing the causes of leave events among Aboriginal and Torres Strait Islander people and evidence-based strategies to reduce them.

## Methods

We followed the reporting guidelines and criteria set in the Preferred Reporting Items for Systematic Review (PRISMA 2020) [11]. A PRISMA checklist demonstrating the recommended items to include in a systematic review was completed and can be found in Supplementary file 2.

### Objectives

1. To understand the factors and causes associated with leave events specific to Aboriginal and Torres Strait Islander people in Australian healthcare settings.
2. To analyse past and current evidenced-based strategies, that have been used to reduce leave events among Aboriginal and Torres Strait Islander people.

### Search strategy

A systematic search was conducted using Medline, Web of Science, Embase and Informit which is a database containing peer-reviewed research with a strong focus on relevant Australian content. We manually searched the webpage of the Australia’s National Institute for Aboriginal and Torres Strait Islander Health Research (Lowitja Institute), Google and Google scholar. We examined the references of the records included to identify possible relevant studies.

The search strategy used key words related to leave events, health services and Aboriginal and Torres Strait Islander people. The search strategy used in each database is available in Supplementary file 3.

### Data extraction

All results were imported to Endnote X9 and duplicates were removed. Screening of titles and abstracts was conducted by CK and JC. Inclusion and exclusion criteria are available in Table 1. Full text of selected records were assessed independently by JC and CK. When available, the following data were extracted by CK from eligible records and organised in an Excel spreadsheet: authors, title, type of document or type of study, journal or place of publication, participants, settings, objectives, and findings. All data were then reviewed by JC by comparing the data entered to the Excel spreadsheet with the results section of the included papers. Discrepancies during the process of screening and data extraction were discussed until consensus was reached.

**Table 1:** Inclusion and Exclusion Criteria

Inclusion Criteria	Exclusion Criteria
1. English language, published from 1990 – 2022. This timeframe was based on time and human resources availability 2. Primary studies including qualitative, quantitative, and mixed methods studies 3. Reports of interventions previously or currently undertaken to reduce leave events among Aboriginal and Torres Strait Islander people 4. Analysis of factors or causes associated with leave events among Aboriginal and Torres Strait Islander people of all ages 5. Studies analysing leave events among Aboriginal and Torres Strait Islander people and other Australians were included when the factors or causes associated with leave events among Aboriginal and Torres Strait Islander people were specifically analysed 6. Analysis of interventions to reduce leave events among Aboriginal and Torres Strait Islander people 7. Analysing leave events in health care services of all levels including hospitalisation and emergency department	1. Studies published in languages other than English 2. Studies including Aboriginal and Torres Strait Islander people and other Australians where causes of leave events were not analysed for Aboriginal and Torres Strait Islander people specifically 3. Studies including routine discharge or negotiated/agreed discharge; discharge for the day programs and instances of ‘did not attend’

### Data analysis

A narrative synthesis was used for quantitative findings [12]. Qualitative studies and reports were analysed following an inductive thematic analysis as described by Braun and Clarke (2006) [13]. Data familiarisation occurred by reading the papers during full-text analysis, then during data extraction and a third time to conduct coding. Coding was conducted by CK (a non-Indigenous researcher) through the identification of the semantic content of every sentence in the results section of each paper. Once codes were identified for each paper, all were collated in a list of codes which were then grouped by CK and JC (an Aboriginal senior researcher) within identified themes. Emerging themes and their conforming codes were then reviewed by all authors. We ensured consideration and respect of Aboriginal and Torres Strait Islander ways of knowing being and doing by engaging a research team led by an Aboriginal woman and comprised mainly by Aboriginal and Torres Strait Islander people (Authors JC, CR, TM, KBB, BP, EB). Aboriginal and Torres Strait Islander authors provided feedback on data analysis and interpretation based on their own knowledges, decolonising research experience and lived experiences. During the data analysis phase, we ensured that the voices of Aboriginal and Torres Strait Islander researchers were prioritised [14].

### Quality assessment

The Mixed Methods Appraisal tool (MMAT) was used to assess the quality of peer reviewed studies [15]. Each study was assessed independently by JC and CK who assigned each paper a score from 0 – 5. The final score was calculated using the average of the reviewer’s scores. Studies were classified as low (0–1), medium (2–3) or high (4–5) quality according to the final score. Quality assessment of the reports using the MMAT was not conducted given heterogenicity of the methods used by each report. A quality assessment of all records from an Aboriginal and Torres Strait Islander perspective, was also conducted by CK and JC using the Aboriginal and Torres Strait Islander quality appraisal tool developed by Harfield et al. (2020) [16]. This tool was used to assess the extent to which included records appropriately conducted community engagement, consultation and used a strength based approach to their research [16].

### Ethical principles

We followed the guidelines from the Australian Institute of Aboriginal and Torres Strait Islander Studies for ethical research in Indigenous studies [17], the guidelines for ethical conduct in Aboriginal and Torres Strait Islander health research (National Health and Medical Research Council, 2018) [18] and the Lowitja’s Institute practical guide for researching Indigenous health [19]. The Aboriginal and Torres Strait Islander Health Program at The George Institute for Global Health [20] have ensured that Indigenous ways of knowing, being and doing were respected throughout the research process.

## Results

**Fig. 1:**
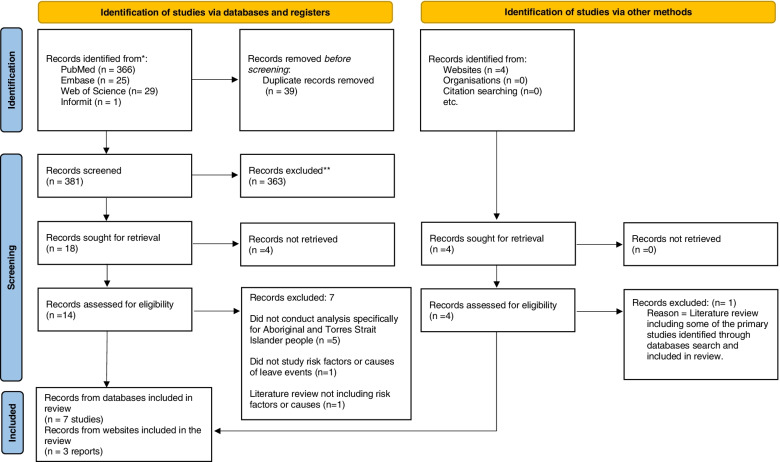
PRISMA 2020 flow diagram for new systematic reviews which included searches of databases, registers and other sources

The initial search located 421 records. After removing duplicates, 381 titles and abstracts were screened from which 18 were selected for full-text assessment. From these, 4 records were conference abstracts, however full reports were not retrieved despite efforts to contact the authors. Only ten of the remaining 14 records met the inclusion criteria. An additional four records were found through Google search but only three met the inclusion criteria. The results of the screening process are depicted in Fig. 1.

A total of ten records were included. Seven were research studies and three were reports describing qualitative data (*n* = 2) [21, 22] and mixed data (*n* = 1) [23]. From the seven research studies, four were quantitative studies (*n* = 4) [24–27] and three were qualitative studies [28–30]. The most recent records were published in 2021 [27, 29, 30] whilst the oldest one is from 2002 [28]. Five records studied data from the Northern Territory [23, 24, 27, 28, 30], two from Western Australia [21, 26], two from New South Wales [22, 25] and one from Queensland [29]. Characteristics of the records are summarised in Table 2.

**Table 2:** General characteristic of the studies

First Author and year of publication	Type of study	Objectives	Data collection methods	Participants and settings	Findings	
Primary Studies					Causes^a^	Recommendations
Einsiedel et al. (2013) [24]	Prospective cohort study	To prospectively identify risk factors for self-discharge Among Aboriginal patients	Individual patient interviews to collect demographic details, understanding of diagnosis and management, alcohol consumption, history of self-discharge, use of traditional healer, social problems, loneliness, and perceptions of the hospital. Univariable and multivariable analysis of risk factors and self-discharge	Aboriginal patients > 14 years in the general medical units at Alice Spring Hospital	-Univariate analysis: Male gender, Age < 45 years, history of self-discharge, desire to drink alcohol, town camp residence, dissatisfaction with treatment and urge to leave hospital -Multivariate analysis: Desire to drink alcohol, Transfer to tertiary centre, past history of self-discharge, male, past history of alcohol dependence	Not studied
Wright (2009) [25]	Comparative retrospective cross-sectional study	Examine difference between Aboriginal and non-Aboriginal people who did not wait to see the medical officer: 1. Examine relationship between triage category assigned and Aboriginal status. 2.Examine relationship between variables did not wait and Aboriginal status 3. Examining relationship between variables discharge against medical advice and Aboriginal status	Data from Emergency Data Information System about Aboriginal status, presenting problem, age, sex, triage category, day and time of presentation, mode of arrival, time waited	All presentations to four emergency departments in the North Coast Area Health Service of NSW between January 2006 and December 2006	-Aboriginal people who did not wait were two times more likely to have arrived by ambulance than non-Aboriginal people -The majority of Aboriginal people who did not wait were young adults (20–24 years) and children -The majority of Aboriginal people who did not wait presented during evening on Monday, Tuesday and Sunday - Strong association between triage 4 (less severe symptoms or injuries) and “did not wait” or “Discharge Against Medical Advice (DAMA)”	Not studied
Katzenellenbogen et al. (2013) [26]	Cross-sectional study	Investigate demographic and clinical factors that predict Discharge Against Medical Advice (DAMA) in patients with first admission for Ischemic Heart Disease with Focus on the differences in risk of DAMA in Aboriginal and non-Aboriginal patients	Data from person-linked file of all admissions to any WA hospital in 1985–2008 with a discharge diagnosis of Ischemic heart disease. Univariate and multivariate logistic regression models to determine predictor of discharge against medical advice and logistic regression modelling applied separately to Aboriginal and non-Aboriginal patients	Aboriginal and non-Aboriginal people admitted to any hospital in WA aged 25–79 years with first-ever admission for Ischemic heart disease	-Aboriginal patients were more likely to DAMA if they had fewer comorbidities -Drug and alcohol dependence was associated with DAMA in Aboriginal and non-Aboriginal patients -Metro hospital and rural residence was associated with DAMA for Aboriginal patients	Not studied
O’ Connor et al. (2021) [27]	Pre-post study	To further explore the likelihood of a causal association between study activities and the decrease in self-discharge rates which occurred during the study intervention period	Linear regression was used to examine the relationship between numbers of interpreter bookings made per month and self-discharge rates	Data was collected from Interpreter bookings between 1 April 2016–31 March 2019 provided by the NT Aboriginal Interpreter Service and leave events were recorded from separations data at the Royal Darwin Hospital	Not studied	Significant inverse association was present between interpreter bookings and likelihood of self-discharge among Aboriginal inpatients
Franks and Beckmann (2002) [28]	Qualitative study	To clarify perceptions of Take Own Leave (TOL) among hospital and community health staff and patients including Definition of TOL, who is likely to TOL, Administrative response to TOL, perceived impact of TOL, Reasons of TOL, proposed solutions to TOL	Semi-structured list of open-ended questions: Interviews with patients and staff of Alice Spring Hospital and two remote Central Australian communities and three focus groups in the hospital with health education staff, medical staff, and nurses at the hospital	Patients, hospital health staff and community health staff of Alice Springs Hospital and two remote Central Australian communities from different language groups	Nurses and doctors speaking rudely, being away from family, heard doctor saying that they were better and though they could go, felt better and did not understand importance of completing treatment, alcohol withdrawal, fear of medical treatment or being send away, previous stories of bad treatment in hospitals and people dying in hospital, sorry business, children or other family at home who need care, job,language barriers, lack of effort from doctors to explain, different perceptions and expectations of health models, unfamiliar hospital environments and lack of understanding of hospital procedures like isolation or restricted diets	More Aboriginal staff specially language speakers, having Aboriginal health worker in each ward, cultural awareness from staff, provide cultural training before start working,community education about hospital environments and procedures, spaces for family visits, access to outdoor spaces, Aboriginal art, more patients receiving treatment at home
Askew et al. (2021) [29]	Qualitative study	To increase understanding about the causative and contextual factors that culminate in people self-discharging and identify opportunities to improve the hospital experience for all	Semi-structured interviews with five Aboriginal and/or Torres Strait Islander people and six non-Indigenous people who had self-discharged from a major tertiary hospital in Brisbane	11 participants (5 Indigenous aged 43–63) from Princess Alexandra Hospital	Use of medical jargon, not enough time taken to explain medical condition, conflicting information from different doctors, perception of staff being rude and alienating, racism, stereotypes, demeaning attitudes, lack of understanding of Aboriginal culture, uncomfortable spaces, responsibilities at home, lack of economic resources for transport and treatment	Not studied
Kerrigan et al. (2021) [30]	Qualitative study	To present Aboriginal language speaking patient experiences and perspectives of hospital care when access to interpreter-mediated communication is consistent	Participant interviews, researcher field notes from shadowing doctors, doctors’ reflective journals, interpreter job logs and patient language lists	Six Aboriginal language speaking patients (five Yolŋu and one Tiwi), three non-Indigenous doctors and five Aboriginal interpreter staff at the Royal Darwin Hospital were purposefully sampled	feeling frustrated and disempowered due to communication issues, use of medical jargon by doctors, responsibilities at home, feeling disrespected by demeaning comments based on stereotypes, uncomfortable hospital spaces, lack of cultural awareness	Use of interpreters increased patient satisfaction and access to services to meet social determinants of health which resulted in reduced self-discharge and re-admissions
Reports						
Aboriginal Health Policy Directorate (2018) [21]	Mixed methods	1. Review relevant policies and recording processes 2. Outline TOL recording and coding pathways 3. Examine rates of TOL in WA 4. Summarise information about contributing factors and impacts received through consultation 5. Provide strategies for improving TOL	Consultation through a template in early 2017 with health service providers, Aboriginal Health Council WA, Health Consumer’s Council, WA Primary health Alliance, Mental Health Commission, and senior WA health staff	Western Australia	Racism and stereotyping, distrust of health services, unwelcoming hospital environments, lack of Aboriginal workforce, Communication and language barriers, family and cultural obligations, social disadvantage, stereotypes about alcohol and drugs, stereotypes about mental health, unstandardised admission and discharge procedures	Cultural competency training for workforce, consultation and partnership to improve coordination, increase use of interpreters, develop culturally appropriate resources, enhance communication with patient and family, create culturally friendly spaces and spaces for family, Increase Aboriginal workforce, address social determinants of health, consider service availability and distance, training staff to reduce stereotyping associated to alcohol and drugs, training to identify and manage intoxicated or withdrawal patients, appropriate assessment and service delivery for mental health conditions, address mental health conditions stigma, culturally appropriate policies procedures and practices throughout pre- admission, admission and discharge, use telehealth to allow patients to communicate with family, Increased understanding of patterns and causes of leave events
Clinical Excellence Commission (2020) [22]	Mixed methods	Report the findings of the "Diagnostic phase " of the NSW Health's Strategic Priority 2.1. were they focused on clinician and expert perspectives about the contributing factors for TOL and how would they like to improve the provision of care for Aboriginal peoples	1. Literature review of peer-reviewed and grey literature 2. Consultation with clinicians and providers in NSW public health facilities 3. Consultation with other national bodies 4. Consultation with industry bodies involved in care of Aboriginal patients 5. Visits to far West local Health District and Western NSW local Health District	New South Wales	Interdependent levels of responsibility: 1. System: improving Aboriginal representation in workforce an appointing Aboriginal leader 2. Organisation: ensuring that patients feel safe, welcomed and understood. 3.Community: establishing relationships with communities to strengthen Aboriginal identity and community control 4. Individual: Need of cultural awareness and sensitivity in workforce and increase patient understanding of their condition and process of care	Shared understanding of the definition of TOL, DAMA, did not wait etc.… Build shared accountability for the appropriate treatment of Aboriginal patients, environments to be more friendly including physical facilities, waiting rooms, Wi-Fi, kid areas, access to water, phone charging. Increase in awareness of importance of family and carers, promote rapport building and shared decision making between practitioners and families, improvement in identification of Aboriginal status, increase representation of Aboriginal staff, more flexible models of care adapted to patient needs, enhancement of relationships between hospital services and primary health services, coordinated approach to measuring and reporting leave events to support learning and improvement
Henry et at. (2007) [23]	Mixed methos	1. “Explore reasons underlying patient self-discharge” 2. “Determine if the behaviour is associated with patient non-compliance and/or resource constrains” 2. “Explore duty of care consequences of self-discharge” 3. “Identify ways to prevent self-discharge” 4. “Assess costs and benefits of options to manage these patients” 5. “Interview patients who had left hospital prematurely”	1. Statistical and economic data analysis from reports of all Northern Territory hospitals between 1999–2004 2. Twelve semi structured Interviews and 6 focus groups with staff in hospitals 3. Six interviews and 1 focus group with Aboriginal Health services and interviews with 30 Aboriginal patients	Royal Darwin Hospital: Key Health service stakeholders including Administrators, doctors, nurses, Aboriginal liaison officers, Aboriginal health workers, Nursing directors, Social Work department, department of Policy Officers and informants of their own hospitalisation or people known to them	Linguistic communication issues, different understanding about disease, treatment and health not respected, use of jargon, discomfort with medical environments and procedures, fear to die in hospital or be referred to other cities, pay bills, send money to family, children at home who need care,sorry business, cultural ceremonies, loneliness, boredom, long periods without family, alcohol and substance withdrawal, transport costs and availability, racism and attitudes from staff	Cultural awareness training, implementation of cultural security and policy procedures in hospitals, patient education about hospital systems, policies, support structures and services available, western concepts of germ and need of fasting. Increase Indigenous staff and their availability including interpreters, flexibility to meet patient needs like exercise, family meetings, bush walks. Information about patient background in the door, better discharge plans, family meetings by telephone, increase support services to meet needs, develop consistent terminology and approaches for identification and recording of self-discharge

^a^All quantitative results reported in the table were statistically significant

### Quantitative findings

Four of the five records (*n* = 4) reporting quantitative findings included patients who self-discharged or discharged against medical advice from hospital units [23, 24, 26, 27] and the other (*n* = 1) patients who did not wait to see a medical officer in an emergency unit [25]. The most commonly measured variables were age and sex (*n* = 4), followed by area of residency (*n* = 3) [23, 24, 26] and socioeconomic status, alcohol use or alcohol related conditions [24, 26] and type of admission (*n* = 2) [23, 26]. Other variables analysed by only one study included understanding of diagnosis, history of self-discharge, use of traditional healer, loneliness, perception of hospital [24], triage category, day and time of presentation, mode of arrival, time waited in Emergency department [25], hospital type, comorbidities [26], medical unit from which self—discharge occurred, season, hospital length of stay [23] and the use of interpreters [27].

Male sex and age of less than 45 years were found to be associated with leave events in three records (*n* = 3) [23, 24, 26]. Two records found an association with town camp residency [23, 24]. Associations with other variables were found only by individual records and included past history of self-discharge, possible transfer to a referral centre, history of alcohol dependence, dissatisfaction with treatment [24], Triage IV allocation [25], admission to hospital as an emergency, history of mental health or alcohol related conditions, fewer comorbidities, and length of hospital stay of approximately 5 days [23]. Only one study found a significant inverse association between interpreter bookings and likelihood of self-discharge among Aboriginal inpatients [27]. No other quantitative data were found analysing strategies or interventions to reduce leave events.

### Qualitative findings

The findings were categorised in two main groups: 1. factors contributing to or causing leave events and 2. suggestions to decrease leave events.

### Causes of leave events

We identified nine themes of causes or factors contributing to leave events. The themes identified and its codes are shown in Supplementary file 4.

#### Intercultural clash and lack of cultural awareness

The understanding of health and healthcare models differs greatly between clinical staff and Aboriginal and Torres Strait lslander people. Clinical staff approached health care and practice from a set knowledge system, one which does not encompass Aboriginal and Torres Strait Islander cultural contexts, practices, worldviews and understandings of health, wellbeing, healing and health care [21, 23, 28, 29]. When clinical teams were not skilled in culturally safe care, patient centred approaches were diminished, resulting in power differentials and marginalisation of patients against their health and wellbeing. Lack of cultural safety led to disagreements and resulted in physician’s being challenged to provide appropriate clinical practice for Aboriginal and Torres Strait Islander patients. This was reported to occur more often among new staff members who did not have experience caring for Aboriginal and Torres Strait Islander patients [23]. For example, participants mentioned that Aboriginal patients who often don’t complain about pain, didn’t receive adequate analgesia by new clinical staff without cultural safety training, as they assumed that the patients were not feeling pain [23].

#### Racism and Stereotypes

Clinical staff perpetuated racism and stereotypes towards Aboriginal and Torres Strait Islander people and devalued their worldviews, practices and health understandings, resulting in inappropriate and discriminatory behaviours [21, 23, 28–30]. Stereotypes and stigma that associates Aboriginal and Torres Strait Islander patients with alcohol consumption, mental health issues and lack of treatment compliance, often resulted in inappropriate assessment, attention, and appropriate treatment options [21, 28, 30].

#### Distrust of health system and fear of unfamiliar environment and procedures

Aboriginal and Torres Strait Islander people felt fear and distrust of the Western health system due to events caused by colonisation, negative past personal experiences with the health system or experiences relayed by a friend or relative [21, 23, 28]. Participants mentioned that hospitals were perceived as unfamiliar environments for Aboriginal and Torres Strait Islander people who often felt fear and anxiety with routine procedures like hospital isolation, waiting for X-rays or fasting before surgery and procedures related to treatment like needles or surgery [23, 28].

#### Lack of availability and unstandardised role of Aboriginal workers

It was reported that Aboriginal workers such as Aboriginal Health Workers (AHW), interpreters and Liaison officers (LOs) were not available all the time since hospitals usually don’t have enough Aboriginal staff available to cover all units, and their shifts cover only standard office working hours despite being needed at all times of the day [21, 23]. Additionally, it was mentioned that the role of AHW workers and LOs is not standardised across hospitals and health settings in Australia which leads to misunderstanding of their function and underutilisation of their skills [21, 23].

#### Communication issues

Communication issues were reported due to language barriers or failure from staff members to deliver information in a clear manner [21, 23, 28–30]. Language barriers were exacerbated when an interpreter was not available and in locations where there was more than one common Aboriginal language [23, 30]. Aboriginal patients often misunderstood or did not understand physicians’ explanations because of the use of medical jargon [23, 29, 30]. Therefore, it was perceived by Aboriginal patients that physicians did not attempt to explain things properly and felt disrespected when spoken to by medical staff [21, 28, 29]. In other cases, physicians assumed that Aboriginal patients who can communicate in English can understand medical jargon [23, 30].

#### Unfriendly hospital environments

Hospitals were perceived as unfamiliar, unfriendly, and uncomfortable spaces. Lack of outdoor areas and places to meet family members, as well as long hospital stays without family visits, led to feelings of fear, anxiety, loneliness, and boredom [21, 23, 28–30]. Additionally, Aboriginal patients felt intimidated and were less likely to make a complaint about staff because everyone was non-Indigenous [23].

#### Social and cultural beliefs and responsibilities

Community is an important aspect of Aboriginal and Torres Strait Islander cultures. Findings showed that Aboriginal and Torres Strait Islander people preferred to stay within their communities and desired to stay in hospital as short as possible. Some patients self-discharged when they were told that they needed to be transferred to another state or city out of fear of not being able to return to Country [23, 28]. This was especially important for people in their last stage of life who feared dying in a hospital and not on “Country” [23, 28]. Many patients self-discharged to attend responsibilities such as paying bills, sending money to their family, caring for other children or sick people at home or cultural obligations like sorry business [21, 23, 28–30]. For example, pregnant women self-discharged because they had other children at home who needed to be looked after [21].

#### Socioeconomic disadvantage

Aboriginal and Torres Strait Islander patients often had to self-discharge due to lack of transport available to return home in remote areas or due to lack of economic resources to pay for transport and other expenses related with hospitalisation [21, 23, 28, 29].

#### Administrative procedures

Some administrative procedures contributed to leave events. These included hospital admission for minor medical conditions which resulted in longer waiting times, unstandardised coding of leave events and procedures to follow-up or notify Aboriginal Medical Services when the events occur, inadequate identification of Aboriginal and/or Torres Strait Islander status and lack of inclusion of the patient and their family in admission and discharge procedures [21, 23, 28].

### Recommendations to decrease leave events

Nine themes were identified as recommendations to decrease leave events among Aboriginal and Torres Strait Islander people. The themes and respective codes are shown in Supplementary file 5.

#### Hospital environment more welcoming and services more friendly to patient cultural needs

The most common theme was adapting hospital environments and services to be more culturally safe and friendly. It was recommended that Aboriginal and Torres Strait Islander leaders and communities should be consulted to guide this process and ensure that Aboriginal and Torres Strait Islander cultural needs are met [21, 22]. Some of the suggested changes include access to outdoor spaces, availability of spaces and more time for gathering with family and friends, more comfortable spaces with access to Wi-fi, phone charging stations, water, areas for children to play, signs and visual aids in Aboriginal and Torres Strait Islander languages to help navigation and provide flexibility to leave the waiting room without missing opportunities for treatment and arrangement of temporal leave when appropriate [21–23, 28]. It was also noted that services need to be adapted to meet cultural needs like women’s and men’s business or fear of dying in hospital [22].

#### Cultural awareness and cultural competency training

Findings suggested the need for provision of ongoing and compulsory cultural capability training for clinical staff [21–23, 28]. Some commented on the need for cultural security policies and procedures in hospitals including a cultural safety committee [21, 23]. Participants commented that the training should include recognition of power imbalances [21], respect for an Aboriginal and Torres Strait Islander concept of health and wellbeing, beliefs, and traditional practices [21]. It was also said that physicians should be trained to engage, build rapport, and improve communication with patients [22]. It was noted that there is a need for medical staff to use plain language when clarifying questions and to provide further information on medical conditions and treatments to improve patient understanding [21, 22].

#### Training for adequate assessment and management of alcohol and drug intoxication and withdrawal and mental health conditions

Participants mentioned the need for providing training to the physicians to reduce stigma and stereotypes that associate Aboriginal and Torres Strait Islander people with alcohol and drug abuse and mental health conditions [21]. Besides providing training to reduce these stereotypes, it was highlighted that there is a need to train in accurate identification and management of patients with alcohol or drug intoxication and withdrawal syndromes and acute mental health conditions [21, 28].

#### Increase number and visibility of Aboriginal health workers

Participants repeatedly suggested increasing the Aboriginal and Torres Strait Islander health system workforce [21–23, 28]. Recommended strategies included to increase the recruitment of Aboriginal Health Workers, translators, Liaison Officers and Aboriginal clinical staff [21, 22, 28]. The qualitative study by Kerrigan et al. reported that access to interpreters resulted in increased patient satisfaction and increased access to social determinants of health which resulted in reduced self-discharge and re-admissions [30]. Further, it was advised that the working hours of AHW, translator and LOs should be expanded to cover operational hours of hospitals [22, 23]. They emphasised the need for role standardisation and specialisation for AHW to ensure that their skills are used timely and in an optimal way [21, 22]. Upskilling non-clinical Aboriginal staff such as Aboriginal Liaison Officers to understand medical language and participate in clinical rounds was also proposed [22]. Other suggestion included increasing recruitment and retention of Aboriginal staff and health related career development pathways for Aboriginal and Torres Strait Islander people [21–23]. More support from allied services such as mental health, drug, and alcohol health services was also suggested to decrease leave events [22, 23].

#### Better communication with patients and patient education about hospital environments and procedures

Another repeated recommendation was improving communication with patients as well as educating about the Western biomedical system and what to expect during hospitalisation [21–23, 28]. The studies emphasised the development of culturally appropriate educational materials, in partnership with Aboriginal and Torres Strait Islander people. These materials should provide information about general Western medical concepts such as germ theory, fasting before surgery and general hospital routines and procedures so that patients have a better understanding of the hospital environment and procedures, and know what to expect to reduce anxiety [21–23]. Two records mentioned the need to improve communication with Aboriginal and Torres Strait Islander patients and their family about triage and the admission process, expected hospital length of stay, medical condition and treatment options, medical procedures, and the discharge process [21, 22]. One record highlighted that communication and informed consent should precede Aboriginal and Torres Strait Islander patients being seen by medical students [21].

#### Involvement of family in health care

Participants also recommended the importance of recognising the pivotal role that family plays within Aboriginal and Torres Strait Islander health and involving family in health care. It was discussed that staff members should engage with families and carers during the health care journey and involve them in the care of the patient and decision-making process [21–23, 28]. Some suggested to use more information technology tools such as teleconferencing to engage and involve family members who are in remote areas [21].

#### Improvement of administrative policy and procedures at hospital and health systems levels

Involvement and accountability of everyone across health systems and organisations, along with the support of Aboriginal and non-Aboriginal leaders, was proposed as essential to build shared accountability and responsibility to decrease leave events [22]. Results emphasised the need of unifying and standardising terminology related to leave events and the procedures to report them and follow-up patients who leave [21, 22]. They stated that discharge processes should be better planned and explained to patients and families from the moment of the admission and should include mechanisms to conduct regular analysis of patient hospital experience, satisfaction, and reasons why they want to self-discharge [21, 22, 28]. Others suggested the arrangement of early discharge when appropriate, the standardisation of admission criteria and outpatient criteria to avoid admission for minor clinical issues, and the utilisation of telehealth to facilitate multidisciplinary management and delivery of health services from home [21, 28].

#### Improve service coordination

Improvement of partnership, communication, and coordination between different levels of health care was advised. Enhancing collaboration and communication between hospital and local community services was proposed to enable the provision of more health care services in the community [21–23]. Participants said that this partnership and coordination should involve the tertiary health care providers, general practitioners, Aboriginal community workers, liaison officers and should include the coordination of services such as outpatient therapy, transport arrangement, and patient education [22, 23].

#### Address socioeconomic factors

Two records stated the need to always consider and address the socioeconomic disadvantages faced by many Aboriginal and Torres Strait Islander people such as lack of transport to remote areas [21, 23]. Examples included arranging transport services as well as providing support with other expenses derived from hospitalisation like accommodation and meals for family members and carers [21, 23].

### Quality assessment

Of the seven studies included, five met high quality criteria [24, 26, 27, 29, 30], and the other two were classified as moderate quality [25, 28]. The results of the quality assessment using the Mixed Methods Appraisal Tool (MMAT) can be found in Supplementary file 6. The results of the quality appraisal from the perspective of Aboriginal and Torres Strait Islander people are shown in Supplementary file 7. Overall, reporting against the Aboriginal and Torres Strait Islander criteria was low. The highest score was achieved by Kerrigan et al. reporting 9 of the 14 criteria.

## Discussion

The quantitative findings of our review demonstrate that leave events occur more often among young Aboriginal patients of male gender, with history of previous leave events and who live in low socioeconomic areas. These are consistent with global literature which have associated leave events with the profile of a young male patient of low socioeconomic status and medical comorbidities related to substance use disorder and mental health disease [2, 31–34]. Unfortunately, research focused on describing the characteristics of patients who leave against medical advice has served to perpetuate the notion that leave events are a deviant behaviour observed in individuals with certain characteristics [35] which can result in patient stigmatisation, and reduced access to care [35, 36]. This deflects the attention from the quality gaps on the health care delivery models that disproportionally impact Aboriginal and Torres Strait Islander people [35, 37].

The qualitative findings of our review revealed in depth some of the causes behind the high representation of leave events among Aboriginal and Torres Strait Islander people and strategies to overcome these. The participants included Aboriginal and Torres Strait Islander patients, Aboriginal and Torres Strait Islander and non-Indigenous health providers and other key health stakeholders, who expressed very similar thoughts related to the factors associated with leave events. Our thematic analysis yielded factors inherent to the provision of health services and to the living context of the patients rather than related to the characteristics of the individuals. These results support other scholars who have argued that leave events can be interpreted as an indicator of health service quality [8, 35, 38].

Robust global evidence has already discussed the urgent need for cultural safety training in health care to reduce racial health disparities that are exacerbated by a dominant Western biomedical system and ongoing institutional racism [39–42]. In Australia the call to implement cultural safety in the healthcare system has been made [43–46] and is well recognised by Commonwealth, State and Territory Governmental bodies [47]. Despite this, the results evidenced that many health practitioners have minimal understanding of Aboriginal and Torres Strait Islander culture and beliefs and show culturally insensitive behaviours [23]. These behaviours seemed to occur especially among new staff members without previous experience caring for Aboriginal and Torres Strait Islander people [23]. This highlights the importance of ensuring continued and ongoing cultural training, along with organisational cultural security policies, procedures, and surveillance to guarantee that services provided are culturally appropriate.

One of the main issues constantly raised was the shortage of Aboriginal and Torres Strait Islander health staff including health workers and liaison officers within the health system [21–23, 28]. AHW play a paramount role in the provision of quality health services for Aboriginal and Torres Strait Islander people [48–50]. AHW provide support by connecting patients to needed services such as accommodation, food and transport which alleviates concerns experienced by patients that result in leave events [49, 50]. Availability of Aboriginal and Torres Strait Islander health workers, liaison officers, doctors, nurses, and other health professionals can reduce the cultural clash between Indigenous and Western systems providing care that meets cultural and spiritual needs. Aboriginal and Torres Strait Islander health staff and interpreters can also improve communication barriers and reduce feelings of fear and anxiety for being in an unfamiliar environment [29, 30]. In 2017 the Aboriginal and Torres Strait Islander Health Workforce working group published the National Aboriginal and Torres Strait Islander Health workforce Strategic Framework 2016–2023 to build a strong and supported Aboriginal and Torres Strait Islander health workforce to provide culturally safe and responsive health care [51]. Outcomes from this review, strongly recommend government, education and training institutions and other stakeholders to refer to the framework and implement and advance urgently needed strategies that promote career development, recruitment and retention of Aboriginal and Torres Strait Islander workforce within the Australian health system.

The results evidenced other characteristics inherent to the way health services are provided to Aboriginal and Torres Strait Islander people that could be improved to reduce the probability of patient leave events. For example, involving the patient and their family in the decision-making process from the moment of admission and providing clear information about the hospitalisation process may help to set expectations and reduce uncertainty and anxiety. Involvement and communication between medical specialists, general practitioners, Aboriginal and Torres Strait Islander health workers, allied health practitioners and community services could lead to better service coordination to enable more care in the community and reduce lengthy hospital stays. Homogenising and standardising terminology related to leave events and procedures to report and follow-up across the Australian health system can facilitate further measurement and development of strategies to reduce leave events.

The World Health Organization (WHO) states that quality of care is critical for achieving universal health coverage. Quality health services must provide patient centred care that responds to individual preferences, needs and values [52]. In this sense, quality health services in Australia must adapt to be sensitive and responsive to the cultural preferences, needs and values of Aboriginal and Torres Strait Islander people. The results suggest the need to redesign facilities to have spaces where Aboriginal and Torres Strait Islander people can gather with family and friends. Services should also be flexible to meet certain cultural needs like women’s and men’s business, fear of dying in hospital and allowing to arrange temporary leave when appropriate to attend culturally important responsibilities such as sorry business [51].

Improving quality of care requires ongoing monitoring and assessment. Patient satisfaction surveys are recognised as useful sources of information to improve quality in healthcare organisations [53]. As suggested by the results, health care services should implement patient satisfaction tools to understand patient’s experience and conduct regular analysis to better identify and improve factors associated with leave events. Quality improvement would be enhanced by developing an incident monitoring system and complaints system that is accessible, culturally safe and encourages and supports Aboriginal and Torres Strait Islander people to participate in monitoring and benchmarking in the key safety and quality issues [54–56].

Quality health services that meet Aboriginal and Torres Strait Islander cultural needs requires compromise and accountability from people across the whole system, using a training system and performance management system to improve service delivery. To meet Aboriginal and Torres Strait Islander cultural needs, it is essential that decision makers work in partnership with Aboriginal and Torres Strait Islander leaders and communities [57].

### Strengths and limitations

Strengths of this review include a systematic and transparent search following standard guidelines, the use of multiple databases, a snowball approach and Google search to include grey literature, and the inclusion of qualitative and quantitative studies and reports.

We conducted a quality assessment of the included studies using a validated and widely used tool. This tool however, was not suitable to evaluate the reports and has not been validated for use in Aboriginal and Torres Strait Islander studies. For this reason, we have integrated the Aboriginal and Torres Strait Islander Quality Appraisal Tool to privilege Aboriginal voices [16, 58]. Overall, the quality of the studies using the MMAT was high. However, all records (studies and reports) failed to meet at least half of the Aboriginal and Torres Strait Islander Quality Appraisal Tool criteria reflecting the need to improve quality and transparency of research with Aboriginal and Torres Strait Islander peoples. The quality of our results and conclusions is limited by the quality of the records included.

Despite the use of different search methods in an attempt to identify as many studies as possible on the topic, our results are limited by the low number of registers retrieved. Furthermore, the search process may have been subject to publication bias. Most of our results were based on qualitative findings which are recognised as not generalisable since its statistical significance cannot be determined [59]. However, we found very similar qualitative findings from studies conducted in Northern Territory, New South Wales, Queensland and Western Australia. This suggests that the quality care gaps associated with leave events Among Aboriginal and Torres Strait Islander people are similar across Australia. Finally, our review included papers discussing strategies that could be implemented to reduce leave events but only two studies evidenced the effectiveness of employing interpreters to reduce leave events [27, 30]. We could not locate any other studies evidencing strategies that were effective in reducing leave events.

## Conclusion

The findings of our systematic review evidenced multiple quality gaps within Australian health care delivery that are associated with leave events among Aboriginal and Torres Strait Islander people. These findings support other academics who argue that leave events should be interpreted as an indicator of health service quality. To reduce leave events, Aboriginal and Torres Strait Islander people should be better represented within the health workforce. In addition, partnership with Aboriginal and Torres Strait Islander leaders and communities is needed within the decision-making process to implement strategies to provide health services that meet Aboriginal and Torres Strait Islander cultural needs. Further research is needed to demonstrate the effectiveness of strategies we have discussed in this systematic review in reducing leave events.

## Supplementary Information

**Additional file 1.**

**Additional file 2.**

**Additional file 3.**

**Additional file 4.**

**Additional file 5.**

**Additional file 6.**

**Additional file 7.**

## Data Availability

The datasets generated and analysed during the current study can be provided upon reasonable request to the corresponding author.

## Abbreviations

AHW: Aboriginal health workers
DAMA: Discharge against medical advice
LOs: Liaison officers
MMAT: Mixed methods appraisal tool
PRISMA: Preferred reporting items for systematic review
WHO: World health organization
