# Supplementary material for: Leave events among Aboriginal and Torres Strait Islander people: a systematic review
Source: BMC Public Health. 2022 Aug 5;22:1488. doi: 10.1186/s12889-022-13896-1 (PMC9354286; doi:10.1186/s12889-022-13896-1)
Supplement: Supplementary file 1 — Additional file 1. [file 12889_2022_13896_MOESM1_ESM.docx]

| Supplementary file 1. Terminology used across States and Territories | | |
| --- | --- | --- |
| State | Source | Terminologies/Definition |
| National | Cultural safety in health care for Indigenous Australians: monitoring framework.  Australian Institute of Health & Welfare, 2019 | Take own leave (TOL) refers to situations where hospital patients choose to leave prior to commencing or completing their treatment.  Incomplete emergency attendances: Patients left at their own risk or did not wait.  Discharge from hospital against medical advice: Patients left against medical advice or were discharged at own risk. |
| Western Australia | Aboriginal Patient Take Own Leave. Review and recommendations for improvement  Department of Health Western Australia, Perth, 2018 | TOL describes the circumstances where a patient chooses to leave prior to commencing or completing treatment. It includes instances where patients in Emergency Department (ED) did not wait (DNW) to receive treatment, abscond or go missing, self-discharge, leave at their own risk (LOR), are away without leave (AWOL) or discharge against medical advice (DAMA).  Did Not Wait: Patients who present to Emergency Services and did not wait to be treated after triage assessment. The patient is given three opportunities to be called at least 10 minutes apart. A DNW is different to DAMA.  Discharge Against Medical Advice: Where a patient leaves against medical advice.  Other TOL events that are recorded as DAMA include:  Away Without Leave: Where a patient takes leave but does not return.  Absconded: Lost or missing patient; and Suspected missing patient: Where a patient cannot be found, has been seen leaving or is suspected of leaving.  If a patient absconds, goes missing or takes leave and does not return from a ward, a Clinical Incident begins, and procedures are followed. The incident is recorded in the WA Health Clinical Incident Management System (CIMS) and the patient’s medical record.  If the patient returns, the incident is cancelled. If the patient cannot be found, the incident is recorded as Discharged Against Medical Advice. |
|  | Admission Policy Reference Manual  Department of Health Western Australia, Perth, 2019 | Discharge Against Medical Advice: When the patient chooses to leave the hospital before the completion of treatment against the advice of the treating medical practitioners. |

| State | Source | Terminologies/Definition |
| --- | --- | --- |
| New South Wales | Diagnostic Report: Understanding contributing factors for Take-Own-Leave in NSW Centre for Aboriginal Health.  Clinical Excellence Commission and NSW Centre for Aboriginal Health, Sydney, 2020 | TOL as an ‘umbrella term’ for describing the occurrence of incomplete medical treatment  Did not wait: Patients who decide not to wait for clinical care to commence or medical assessment following triage in the emergency department.  Discharge against medical advice: Patients who have been admitted to hospital who leave against the expressed advice of their treating physician.  Left at own risk: Any person who leaves against advice after treatment has commenced. A diagnosis is required. Includes those patients who were planned for admission but who did not physically leave the ED to another treatment location prior to their departure. |
|  | Mid-term evaluation of the NSW Aboriginal Health plan 2013-2023.  NSW Health, 2019. | Incomplete ED visits: visits for which the patient either left the ED before receiving a medical assessment or left the ED after a medical assessment but before completion of care or ED discharge. |
| Northern Territory | Key performance indicators 2017/18 – attribute sheets.  DOH Northern Territory Govt, 2017 | Discharge/leave against medical advice within 48 hours (DAMA/LAMA): patients who leave/discharge themselves from hospital against medical advice. |
|  | An evidence-based approach to reducing discharge against medical advice amongst Aboriginal and Torres Strait Islander patients.  Deeble Institute, 2016. | Discharge against medical advice (DAMA) is defined as the occurrence of an in-patient leaving a hospital or healthcare setting before discharge is advised by the treating provider. DAMA is also referred to as self-discharge, absconding, taking own leave (TOL) and away without leave (AWOL). |

| State | Source | Terminologies/Definition |
| --- | --- | --- |
| Tasmania | Tasmanian Admitted Patient Dataset - 2019.  AIHW, 2019 | CODE Z Left against medical advice: The code is used for patients who have discharged themselves against their doctor’s advice. |
| South Australia | Aboriginal health in South Australia: 2017 case study.  Health Performance Council, Govt of South Australia, 2017. | Inpatient discharge against medical advice: Public hospital inpatients who discharge themselves early against medical advice.  Left emergency department at own risk: persons who prematurely leave public hospital emergency departments (EDs) at their own risk after treatment has already commenced. |
| Victoria | Section 3 – Data Definitions, Victorian Admitted Episodes Dataset (VAED) manual 2019- 20.  Victoria State Government, 2019. | **Left against medical advice**: Patient absconds or leaves against medical advice, at own risk. This includes newborns taken from the hospital against medical advice. |
| Queensland | Queensland Hospital Admitted Patient Data Collection (QHAPDC) Manual 2019- 2020.  Queensland State Government, 2019. | **Code 07 Discharged at own risk**: Patients who abscond or leave hospital against medical advice. |
| Australian Capital Territory | ACT Public Health Services Quarterly Performance Report – Technical and Supplementary Information Quarter 2 2019-20.  ACT Health, 2019. | **Patient who did not wait to be seen**: Patient who did not wait to be seen by a health care professional. |
