# Supplementary material for: Leave events among Aboriginal and Torres Strait Islander people: a systematic review
Source: BMC Public Health. 2022 Aug 5;22:1488. doi: 10.1186/s12889-022-13896-1 (PMC9354286; doi:10.1186/s12889-022-13896-1)
Supplement: Supplementary file 3 — Additional file 3. [file 12889_2022_13896_MOESM3_ESM.docx]

Supplementary file 3. Search Strategy

PubMed:

(("Health Services"[MeSH Terms] OR "Emergency Medical Services"[MeSH Terms] OR "Hospitals"[MeSH Terms] OR "Primary Health Care"[MeSH Terms] OR "health services, indigenous"[MeSH Terms] OR "Aboriginal health services"[All Fields] OR "Community health services"[All Fields] OR "Aboriginal medical services"[All Fields] OR "Primary health care services"[All Fields] OR "Tertiary care"[All Fields] OR (("acute"[All Fields] OR "acutely"[All Fields] OR "acutes"[All Fields]) AND ("Health Services"[MeSH Terms] OR ("health"[All Fields] AND "services"[All Fields]) OR "Health Services"[All Fields] OR ("health"[All Fields] AND "service"[All Fields]) OR "health service"[All Fields]) AND ("organisation"[All Fields] OR "organization and administration"[MeSH Subheading] OR ("organization"[All Fields] AND "administration"[All Fields]) OR "organization and administration"[All Fields] OR "organization"[All Fields] OR "organizations"[MeSH Terms] OR "organizations"[All Fields] OR "organisation s"[All Fields] OR "organisational"[All Fields] OR "organisations"[All Fields] OR "organise"[All Fields] OR "organised"[All Fields] OR "organiser"[All Fields] OR "organisers"[All Fields] OR "organises"[All Fields] OR "organising"[All Fields] OR "organization s"[All Fields] OR "organizational"[All Fields] OR "organizations s"[All Fields] OR "organize"[All Fields] OR "organized"[All Fields] OR "organizer"[All Fields] OR "organizer s"[All Fields] OR "organizers"[All Fields] OR "organizes"[All Fields] OR "organizing"[All Fields])) OR "Aboriginal community-controlled health services"[All Fields] OR "Hospital"[All Fields] OR "clinic*"[All Fields] OR "outpatient*"[All Fields] OR "Local health network"[All Fields] OR "Local health district"[All Fields] OR "Primary Health Network"[All Fields]) AND ("Treatment Refusal"[MeSH Terms] OR "Patient Dropouts"[MeSH Terms] OR (("leave"[All Fields] OR "leaved"[All Fields] OR "leaving"[All Fields] OR "plant leaves"[MeSH Terms] OR ("plant"[All Fields] AND "leaves"[All Fields]) OR "plant leaves"[All Fields] OR "leaves"[All Fields]) AND ("event"[All Fields] OR "event s"[All Fields] OR "events"[All Fields])) OR "discharge against medical advice"[All Fields] OR ("take"[All Fields] AND "own"[All Fields] AND ("leave"[All Fields] OR "leaved"[All Fields] OR "leaving"[All Fields] OR "plant leaves"[MeSH Terms] OR ("plant"[All Fields] AND "leaves"[All Fields]) OR "plant leaves"[All Fields] OR "leaves"[All Fields])) OR "Absent without leave"[All Fields] OR "did not wait"[All Fields] OR ("left"[All Fields] AND "own"[All Fields] AND ("risk"[MeSH Terms] OR "risk"[All Fields])) OR "left against medical advice"[All Fields] OR "discharge at own risk"[All Fields] OR ("away"[All Fields] AND ("leave"[All Fields] OR "leaved"[All Fields] OR "leaving"[All Fields] OR "plant leaves"[MeSH Terms] OR ("plant"[All Fields] AND "leaves"[All Fields]) OR "plant leaves"[All Fields] OR "leaves"[All Fields])) OR "self discharge"[All Fields] OR "Treatment Refusal"[All Fields] OR "Patient Dropouts"[All Fields] OR "refusal to participate"[All Fields] OR (("divorce"[MeSH Terms] OR "divorce"[All Fields] OR "separated"[All Fields] OR "separation"[All Fields] OR "separations"[All Fields] OR "separabilities"[All Fields] OR "separability"[All Fields] OR "separable"[All Fields] OR "separate"[All Fields] OR "separately"[All Fields] OR "separates"[All Fields] OR "separating"[All Fields] OR "separational"[All Fields] OR "separative"[All Fields] OR "separator"[All Fields] OR "separators"[All Fields]) AND ("Health Services"[MeSH Terms] OR ("health"[All Fields] AND "services"[All Fields]) OR "Health Services"[All Fields])) OR "frequent presenters"[All Fields] OR "revolving door"[All Fields]) AND "english"[Language] AND (("indige*"[All Fields] OR "aborigin*"[All Fields] OR "Torres Strait Islander"[All Fields] OR "First Nations"[All Fields] OR "First People"[All Fields] OR "Native Australians"[All Fields] OR "First Australians"[All Fields]) AND "english"[Language])) AND ((english[Filter])

Embase:

1. indigenous people/ or *indigenous australian/ or first nation/ or "Torres Strait Islander".mp. or Aboriginal.mp. or "first people".mp. [mp=title, abstract, heading word, drug trade name, original title, device manufacturer, drug manufacturer, device trade name, keyword, floating subheading word, candidate term word]
2. hospital department/ or private hospital/ or hospital/ or general hospital/ or hospital service/ or community hospital/ or rural hospital/ or public hospital/ or emergency health service/ or emergency care/ or hospital emergency service/ or health service/ or medical service/ or health service/ or primary health care/ or community care/ or "aboriginal medical service".mp. or "aboriginal community controlled health services".mp. or "acute health service".mp. or hospital.mp. or clinic*.mp. or "local health network".mp. or "local health district".mp. [mp=title, abstract, heading word, drug trade name, original title, device manufacturer, drug manufacturer, device trade name, keyword, floating subheading word, candidate term word]
3. patient dropout/ or "leave events".mp. or "discharge against medical advice".mp. or "take own leave".mp. or "absent without leave".mp. or "did not wait".mp. or "left at own risk".mp. or "left against medical advice".mp. or "discharge at own risk".mp. or "away without leave".mp. or "self-discharge".mp. or “self-discharge “or "refusal to participate".mp. or "separation from health services".mp. [mp=title, abstract, heading word, drug trade name, original title, device manufacturer, drug manufacturer, device trade name, keyword, floating subheading word, candidate term word]
4. 1 and 2 and 3
5. limit 4 to (english language)

Web of Science:

1. TS=(Indige* OR Aborigin* OR "Torres Strait Islander" OR "First Nations" OR "First People" OR "Native Australians" OR "First Australians" OR native)
2. TS=(“Health care” OR “Aboriginal health services” OR “Community health services” OR “Aboriginal medical services” OR “Primary health care services” OR “Tertiary care” OR “Acute health service organisations” OR “Aboriginal community-controlled health services” OR “Hospital” OR Clinic* OR Outpatient* OR “Local health network” OR “Local health district” OR “Primary Health Network” OR “Emergency department”)
3. TS=("Leave events" OR "discharge against medical advice" OR "Take own leave" OR "Absent without leave" OR "did not wait" OR "left at own risk" OR "left against medical advice" OR "discharge at own risk" OR "away without leave" OR "self discharge" OR "self-discharge" OR "treatment refusal" OR "patient dropouts" OR "refusal to participate" OR "separation from health services" OR "frequent presenters" OR "revolving door" )
4. (#3 AND #2 AND #1) AND LANGUAGE:(English)
