# Supplementary material for: Leave events among Aboriginal and Torres Strait Islander people: a systematic review
Source: BMC Public Health. 2022 Aug 5;22:1488. doi: 10.1186/s12889-022-13896-1 (PMC9354286; doi:10.1186/s12889-022-13896-1)
Supplement: Supplementary file 4 — Additional file 4. [file 12889_2022_13896_MOESM4_ESM.docx]

Supplementary file 4. Causes of Leave Events.

| Theme | Codes |
| --- | --- |
| 1. Intercultural clash and lack of cultural awareness (21,23,28,29). | - Medical staff have minimum understanding of Indigenous mothering and ways of children upbringing (23). - Different understanding about disease, medical treatment, health, and wellbeing and health care model (23,28). - Staff members do not understand Aboriginal people contexts and difficult choices they must make (21,23,28,29). - Culturally, some male patients feel uncomfortable being spoken or cared by female doctors (23). - New staff without knowledge and experience of caring for Aboriginal patients (23). - Lack of appropriate cultural training leads to problems like not giving analgesia to Aboriginal patients who often don’t complain about pain (23). - Is hard for physicians to differentiate between non-compliant patients and patients with socio-economic issues (23). - Patients are unhappy with medical treatment received (23,28). - Disagreement about treatment regimen (23, 28). |
| 1. Racism and stereotypes (21,23, 28,29,30). | - There is a belief that Aboriginal and Torres Strait Islander mothers are not good and are made to feel uncomfortable (23,29). - Special care nursery is perceived as a hostile and alienating environment (23). - Staff devaluate Aboriginal and Torres Strait Islander worldviews and health understanding (23). - Inappropriate and insensitive behaviour or lack of attention from staff members towards Aboriginal and Torres Strait Islander patients (23). - Patients feel upset about the way they are treated or spoken to (23,28,29). - All treatment options are not explored because of stereotype of Aboriginal people being non-compliant (21,29,30). - Covert racism and implicit bias affecting medical decisions and behaviours toward Aboriginal and Torres Strait Islander patients (21,29,30). - Stereotyping and stigma associated with mental health conditions leading to inappropriate assessment, attention, or counselling (21). - Discrimination and stereotyping related to alcohol consumption and mental diseases (21,28). - Lack of appropriate alcohol withdrawal assessment and management (21, 28). - Assumption that patient is intoxicated by alcohol or drugs (21,28). |
| 1. Distrust of health system and fear of unfamiliar environment and procedures (21,23, 28). | - People fear authorities and repercussions for bringing sick or injured children to hospital (23). - Patients feel fear and anxiety with many medical procedures like needles, isolation, long waiting times, surgery (23). - Some family members get worried and want to take the person home (23). - Many people don’t understand why they are in hospital and what is going to happen to them (23,28). - Aboriginal patients have the perception that hospital is a place where people go to die (23). - Reduced trust in the system due to racism and stereotyping (21). - Historical, personal, and family negative experiences with health systems leading to distrust (21 , 28) - Fear of medical treatment (28). - Lack of understanding of hospital environment and procedures (28). |
| 1. Lack of availability and unstandardised role of Aboriginal workers (21,23). | - They are more likely to self-discharge when Aboriginal Liaison officers are not at work (23). - Aboriginal Health Workers are not able to use their skills in a hierarchical hospital system (23). - Aboriginal Health Workers are not used as brokers and are called after problems have occurred and patient self-discharged (23). - No availability of interpreters in all units (21,23). - Aboriginal Health Workers and interpreters available only during standard working hours (21,23). - Not enough Aboriginal staff available (21). - Inconsistent policies and understandings of the role of Aboriginal Health Workers (21). |
| 1. Communication issues (21,23,28,29,30). | - Use of medical jargon and technical language contributing to non-understanding of medical condition and treatment (23,28,29,30). - Doctors assume that Indigenous patients who can speak English can understand everything (23). - Difficulties with many different Indigenous languages (21,23,28,30). - Miscommunication leading to confusion and perception of staff being disrespectful (21,29). - Patients though it was okey to go (28,30). - Lack of effort from staff members to explain (28,29). - Lack of awareness about need to sign summary discharge (28,30). |
| 1. Unfriendly hospital environments (21,23,28,29,30). | - Uncomfortable hospital spaces without outdoor areas or spaces to meet family (23,29,30). - Aboriginal patients feel intimidated to make complains because everyone is non-Indigenous (23). - Unfamiliar and unwelcoming hospital environments leading to fear and anxiety (21,28). - Isolation, loneliness, and boredom of patients from remote areas or with long hospital stays (23,28). |
| 1. Social and cultural beliefs and responsibilities (21,23,28,29,30). | - Some women are humbugged or intimidates by family or community members and leave (23). - Children or other sick people at home (21,23,28,29,30). - Need to attend responsibilities like paying bills or sending money to family (23,28). - They self-discharge to attend to cultural obligations like sorry business (23,28). - Patients don’t want to stay in hospital for long time and leave when they feel better (23). - Patients are afraid of dying in hospital and not in Country (23,28). - Fear of being transferred to another state or city (23,28). |
| 1. Socioeconomic disadvantage (21,23,28,29). | - Lack of public transport or economic resources available to return home (21,23,29). - Decision to leave when a private car is going to their remote community (23,28). - Lack of economic resources to pay for it or for other expenses related with hospitalisation (21,29). |
| 1. Administrative procedures (21,23,28). | - Admission for minor issues with consequent longer waiting periods (21). - Confusion about coding of leave events (21). - Lack of inclusion of patient and family in admission and discharge procedures (21). - Long waiting times (21). - Variable follow-up procedures (28). - There are no formal arrangements to follow up or notify Aboriginal Medical Services when patients self-discharge (23). - Lack of reliability in identification of Indigenous patients due to discomfort in asking, fear of offending by asking and not looking Aboriginal (28). |
