# Supplementary material for: Leave events among Aboriginal and Torres Strait Islander people: a systematic review
Source: BMC Public Health. 2022 Aug 5;22:1488. doi: 10.1186/s12889-022-13896-1 (PMC9354286; doi:10.1186/s12889-022-13896-1)
Supplement: Supplementary file 5 — Additional file 5. [file 12889_2022_13896_MOESM5_ESM.docx]

Supplementary file 5. Recommendations to reduce leave events.

| Theme | Codes |
| --- | --- |
| 1. Hospital environment more welcoming and services more friendly to patient cultural needs (21,22,23,28) | - Hospital environment with outdoor spaces and spaces for gathering with family (21,23,28). - Allow for family visits, and activities like shopping (28). - Improvement of spaces like more comfortable chairs, Wi-Fi, phone charging stations, water, and areas for children (22,28). - Flexibility to leave waiting room without missing opportunity for treatment (22). - Signs in Aboriginal local language and Aboriginal artwork (22,23). - Spaces for Aboriginal gathering (21,22,23,28). - Adaptation of services to meet cultural needs like women’s and men’s business, fear of dying in hospital (22). - Culturally safe hospital spaces with input of Aboriginal communities (21,22). - Culturally appropriate signs and visual aids to facilitate navigation (21,23). - Providing activities to avoid loneliness and boredom (28). - Arranging temporary leave when appropriate (28). - Balancing medical treatment and patient needs (22,28). |
| 1. Cultural awareness and cultural competency training (21,22,23,28) | - Cultural awareness training for staff before starting work (28). - Cross-cultural training for staff (23,28) - Continued and extended training in cultural awareness with training delivered more often (23,28). - Implementation of cultural security policy and procedures in hospitals including a cultural safety committee (23). - Training to staff about building rapport and engaging better with patients (21, 22, 23). - Training staff to use of plain language and clarification of questions and information (22,23). - Mandatory Cultural competency training (21) . - Continuous review of cultural competency training and assessment of effectiveness (21). - Understand distrust and fear (21). - Recognise power imbalances and take time to build trust (21). - Recognising and respecting Indigenous health concepts, beliefs and traditions (21). |
| 1. Training for adequate assessment and management of alcohol and drug intoxication and withdrawal and mental health conditions (21, 28). | - Education to address stereotype related to alcohol consumption (21, 28). - Training to identify and manage patients with alcohol and drug intoxication and withdrawal (21) - Appropriate assessment and management of mental health conditions (21) - Training to address stereotyping related with mental health disease (21) - Improve management of alcohol withdrawal (28). |
| 1. Increase number and visibility of Aboriginal Health Workers (21, 22, 23, 28,30). | - More visible Indigenous staff (23,28). - Upskilling of non-clinical staff like AHW to understand medical language and participate in clinical rounds (22). - More support from allied services such as Health Workers, mental health, drug, and alcohol services (23,28). - Promote careers, study and professional development for Indigenous Aboriginal Health Workers, doctors and nurses (21, 22,23). - More availability and use of interpreters and service navigators (21,22,30). - Early use and intervention of Aboriginal Health Workers (22). - Increased retention of Aboriginal staff (22, 23, 28). - Role specialisation and standardisation of Aboriginal Health Workers (22,). - Funding for AHW roles in rural areas (22). - Expand working hours of AHW to 28h operating hours of hospitals (22,23). - Engagement of Aboriginal stakeholders to ensure that cultural needs are being holistically addressed (21,22, 23). - Increased employment of Aboriginal Health Workers and liaison officers (21,22,23,28). - Support attraction, recruitment, development, and retention of Aboriginal workforce at all levels (21). - Consistent scope and practice of AHW roles (21). - Place intercultural staff in each ward (21, 28). |
| 1. Better communication with patients and patient education about hospital environments and procedures (21, 22, 23, 28). | - Patient education about hospital environment, what happens in hospital and what to expect during hospitalisation (22,23,28). - Education about services available for them (23). - Patient education about western germs theory and importance of fasting for surgery (23). - Consultation with experts to develop educational material for Indigenous patients (21,23). - Culturally appropriate informative tools and resources (21,22). - Address concerns and expectations of patients (21). - Clear communication and informed consent to be seen by medical students (21). - Improve health literacy through education activities (21,22,23). - Allay fears about procedures (21,22,28). - Resolving conflict between patients and staff member rapidly (22,28). |
| 1. Involvement of family in health care (21, 22, 23, 28). | - Involvement of family though telephone to explain health needs and empower patient and his family (23). - Person-centred care approach focusing on engagement with patient, family, and carers (22). - Health services responsive to individual values and preferences (21,22). - Building rapport with patient, family and carers and involving them in the decision-making process (22). - Recognise and respect role of family in Aboriginal wellbeing (21). - Use of IT to allow patient contact and communication with family (21). - Allow family contact and visits (21, 23, 28). |
| 1. Improvement of administrative policy and procedures at hospital and health systems levels (21, 22 28). | - A shared understanding of terminology related to leave events to provide common language for discussion, measurement, and strategies (22). - Involvement and distributed accountability and responsibility across system and organisations (22). - Leadership of Aboriginal and non-Aboriginal people to build shared accountability and responsibility (22). - Implementation of discharge processes that explore patient needs, satisfaction, and rationale for self-discharge (22). - Implement mechanisms to request leave (22). - Discharge planning should start from admission by setting expectations and enabling care in the community when appropriate (22). - Improvement in the quality of reporting leave events (22). - Improve rate of patient survey completion (21,22). - Implement culturally appropriate admission and discharge policies and procedures(21). - Consistency of admission criteria and outpatient treatment criteria (21). - Standardise follow-up procedures when a patient self-discharge (21,22). - Adoption of telehealth to allow multidisciplinary management and avoid unnecessary travel (21). - Conduct regular analysis to better identify and understand factors associated with leave events (21,22,28). - Improve patient experience tools to understand patient’s experience (21). - Arrangement of early discharge (28). - Increase treatment from home (28). |
| 1. Improve service coordination (21,22,23). | - Better discharge plans involving GPs, community health clinics for ongoing care (23). - Coordination of services needed like transport and outpatient therapy (23). - Support for workforce, education, and partnership (22). - Collaboration, communication and coordination between hospital and community services to enable more care in the community (21,22,23). |
| 1. Address socioeconomic factors (21,23) | - Arrange transport to patients in remote areas (21,23). - Consider availability of transport and local services (21). - Provide support with transport and accommodation- related expenses when necessary (21,23). |
