# Supplementary material for: Leave events among Aboriginal and Torres Strait Islander people: a systematic review
Source: BMC Public Health. 2022 Aug 5;22:1488. doi: 10.1186/s12889-022-13896-1 (PMC9354286; doi:10.1186/s12889-022-13896-1)
Supplement: Supplementary file 6 — Additional file 6. [file 12889_2022_13896_MOESM6_ESM.docx]

Supplementary file 6. Quality assessment using the Mixed Methods Appraisal Tool (MMAT).

| Study | Category of study design | Score Reviewer 1 | Score Reviewer 2 | Average |
| --- | --- | --- | --- | --- |
| Einsiedel et al (2013) (24) | Quantitative non-randomized | 4 | 4 | 4 |
| Wright (2009).  (25) | Quantitative non-randomized | 3 | 3 | 3 |
| Katzenellenbogen et al (2013).  (26) | Quantitative non-randomized | 5 | 5 | 5 |
| O’Connor et al (2021) (27) | Quantitative non-randomized | 4 | 4 | 4 |
| Franks and Beckmann (2002).  (28) | Qualitative | 3 | 2 | 2.5 |
| Askew et al (2021) (29) | Qualitative | 5 | 5 | 5 |
| Kerrigan et al (2021) (30) | Qualitative | 5 | 5 | 5 |

Low Quality: 0-1

Moderate Quality: 2-3

High Quality: 4-5
