# Supplementary material for: Leave events among Aboriginal and Torres Strait Islander people: a systematic review
Source: BMC Public Health. 2022 Aug 5;22:1488. doi: 10.1186/s12889-022-13896-1 (PMC9354286; doi:10.1186/s12889-022-13896-1)
Supplement: Supplementary file 7 — Additional file 7. [file 12889_2022_13896_MOESM7_ESM.docx]

| Supplementary file 7. Quality appraisal using The Aboriginal and Torres Strait Islander Quality Appraisal Tool | | | | | | | | | | | | | | | | | | | | | | | | | | | | | | |  | | | | | |  | | | | | |  | | | | |
| --- | --- | --- | --- | --- | --- | --- | --- | --- | --- | --- | --- | --- | --- | --- | --- | --- | --- | --- | --- | --- | --- | --- | --- | --- | --- | --- | --- | --- | --- | --- | --- | --- | --- | --- | --- | --- | --- | --- | --- | --- | --- | --- | --- | --- | --- | --- | --- |
| Question | Einsiedel (2013)  (24) | | | | Wright (2009) (25) | | | | Katzenellenbogen  (2013) (26) | | | | Franks (2002) (28) | | | | Department of Health WA (2018) (21) | | | | NSW Government  (2020) (22) | | | | Henry (2007) (23) | | | | | O’Connor (2021) (27) | | | | | | Askew (2021) (29) | | | | | | Kerrigan (2021) (30) | | | | | |
| Y: yes P: partially N:No U: Unclear | Y | P | N | U | Y | P | N | U | Y | P | N | U | Y | P | N | U | Y | P | N | U | Y | P | N | U | | Y | P | N | U | | | Y | P | N | U | | | Y | P | N | U | | | Y | P | N | U |
| 1. Did the research respond to a need or priority determined by the community? |  |  | x |  |  |  |  | x |  | x |  |  |  |  |  | x | x |  |  |  |  |  |  | x | |  | x |  |  | | |  | x |  |  | | |  | x |  |  | | | x |  |  |  |
| 2.Was community consultation and engagement appropriately inclusive? |  |  |  | x |  |  |  | x |  |  |  | x |  |  |  | x | x |  |  |  |  | x |  |  | | x |  |  |  | | |  | x |  |  | | |  | x |  |  | | |  | x |  |  |
| 3.Did the research have Aboriginal and Torres Strait Islander leadership? | x |  |  |  |  |  |  | x |  |  |  | x | x |  |  |  |  |  |  | x |  |  |  | x | |  |  |  | x | | | x |  |  |  | | | x |  |  |  | | | x |  |  |  |
| 4.Did the research have Aboriginal and Torres Strait Islander governance? |  |  |  | x |  |  |  | x |  |  |  | x |  | x |  |  |  | x |  |  |  |  |  | x | |  | x |  |  | | |  | x |  |  | | |  |  |  | x | | |  |  |  | x |
| 5.Were local community protocols respected and followed? |  | x |  |  |  |  |  | x |  |  |  | x |  | x |  |  |  |  |  | x |  |  |  | x | |  |  |  | x | | |  |  |  | x | | |  |  |  | x | | | x |  |  |  |
| 6.Did the researchers negotiated agreements in regard to rights of access to Aboriginal and Torres Strait Islander people’s existing intellectual and cultural property? |  |  |  | x |  |  |  | x |  |  |  | x |  |  |  | x |  |  |  | x |  |  |  | x | |  |  |  | x | | |  |  |  | x | | |  |  |  | x | | |  |  |  | x |
| 7.Did the researchers negotiate agreements to protect Aboriginal and Torres Strait Islander ownership of intellectual and cultural property created through research? |  |  |  | x |  |  |  | x |  |  |  | x |  |  |  | x |  |  |  | x |  |  |  | x | |  |  |  | x | | |  |  |  | x | | |  |  |  | x | | |  |  |  | x |
| 8.Did Aboriginal and Torres Strait Islander peoples and communities have control over the collection and management of research materials? |  |  |  | x |  |  |  | x |  |  |  | x |  |  |  | x |  |  |  | x |  |  |  | x | |  |  |  | x | | |  |  |  | x | | |  |  |  | x | | |  |  |  | x |
| 9.Was the research guided by an Indigenous research paradigm ? |  |  | x |  |  |  |  | x |  |  | x |  |  |  |  | x |  |  |  | x |  |  |  | x | |  |  |  | x | | |  | x |  |  | | |  | x |  |  | | | x |  |  |  |
| 10. Does the research take a strengths-based approach, acknowledging and moving beyond practices that have harmed Aboriginal and Torres Strait Islander peoples in the past? |  |  | x |  |  | x |  |  |  | x |  |  | x |  |  |  | x |  |  |  | x |  |  |  | | x |  |  |  | | | x |  |  |  | | | x |  |  |  | | | x |  |  |  |
| 11.Did the researchers plan and translate the findings in policy and/or practice? |  |  |  | x |  |  | x |  |  |  |  | x |  |  |  | x |  | x |  |  | x |  |  |  | |  |  |  | x | | | x |  |  |  | | | x |  |  |  | | | x |  |  |  |
| 12. Did the research benefit the participants and Aboriginal and Torres Strait Islander communities? |  |  | x |  |  |  | x |  |  |  | x |  |  |  | x |  |  |  | x |  |  |  | x |  | |  |  | x |  | | | x |  |  |  | | | x |  |  |  | | | x |  |  |  |
| 13.Did the research demonstrate capacity strengthening for Aboriginal and Torres Strait Islander individuals? |  | x |  |  |  |  | x |  |  |  | x |  |  | x |  |  |  |  |  | x |  |  |  | x | |  |  |  | x | | | x |  |  |  | | | x |  |  |  | | | x |  |  |  |
| 14. Did everyone involved in the research have opportunities to learn from each other? |  | x |  |  |  |  |  | x |  |  |  | x |  | x |  |  |  |  |  | x |  |  |  | x | |  |  |  | x | | |  |  |  | x | | |  | x |  |  | | | x |  |  |  |
